# Diagnostic Value of Contrast-Enhanced Ultrasound for Evaluation of Transjugular Intrahepatic Portosystemic Shunt Perfusion

**DOI:** 10.3390/diagnostics11091593

**Published:** 2021-09-01

**Authors:** Constantin A. Marschner, Thomas Geyer, Matthias F. Froelich, Johannes Rübenthaler, Vincent Schwarze, Dirk-André Clevert

**Affiliations:** 1 Department of Radiology, University Hospital, LMU Munich, 81377 Munich, Germany; thomas.geyer@med.uni-muenchen.de (T.G.); Johannes.ruebenthaler@med.uni-muenchen.de (J.R.); Vincent.Schwarze@med.uni-muenchen.de (V.S.); Dirk.Clevert@med.uni-muenchen.de (D.-A.C.); 2 Department of Clinical Radiology and Nuclear Medicine, University Medical Centre Mannheim, 68167 Mannheim, Germany; matthias.froelich@umm.de

**Keywords:** contrast-enhanced ultrasound, transjugular intrahepatic portosystemic shunt, color doppler ultrasound, computed tomography, magnetic resonance tomography

## Abstract

Background: In patients with liver cirrhosis, transjugular intrahepatic portosystemic shunt (TIPS) displays an effective method for treating portal hypertension. Main indications include refractory ascites and secondary prevention of esophageal bleeding. Color Doppler ultrasound (CDUS) plays a leading role in the follow-up management, whereas contrast-enhanced ultrasound (CEUS) is not routinely considered. We compared the efficacy of CEUS to CDUS and highlighted differences compared to findings of corresponding computed tomography (CT) and magnetic resonance imaging (MRI). (2) Methods: On a retrospective basis, 106 patients with CEUS examination after TIPS were included. The enrollment period was 12 years (between 2008 and 2020) and the age group ranged from 23.3 to 82.1 years. In addition, 92 CDUS, 43 CT and 58 MRI scans were evaluated for intermodal comparison. (3) Results: Intermodal analysis and comparison revealed a high level of concordance between CDUS, CT and MRI in the vast majority of cases. In comparison to CDUS, the correlation of the relevant findings was 92.5%, 95.3% for CT and 87.9% for MRI. In some cases, however, additional information was provided by CEUS (4) Conclusions: CEUS depicts a safe and effective imaging modality for follow-up after TIPS. In addition to CDUS, CEUS enables specific assessment of stent pathologies and stent dysfunction due to its capacity to dynamically visualize single microbubbles at high spatial and temporal resolution. Due to the low number of adverse events regarding the application of contrast agents, CEUS can be administered to a very broad patient population, thus avoiding additional radiation exposure compared to CT angiography in cases with divergent findings during follow-up.

## 1. Introduction

The origin of transjugular intrahepatic portosystemic shunt (TIPS) was in 1969, when Josef Rosch was attempting to find a transjugular way to invasively visualize the bile duct system in an animal experiment for. The idea of portal vein decompression by creating a way to bypass the liver was developed through an unintentional puncture of the portal vein in his experiments [1]. The first clinical application with a balloon dilatated intrahepatic portosystemic shunt followed in 1982 in patients with liver cirrhosis and variceal bleeding [2]. The first clinical application with an expandable metal stent to treat portal hypertension was followed in 1988 by Martin Rossle et al. from Freiburg, Germany [3].

Nowadays, TIPS is an effective treatment option that can successfully handle the complications of portal hypertension in cirrhotic patients [4,5]. The two main indications for TIPS are secondary prevention of esophageal bleeding and refractory ascites while the main adverse event after TIPS involves new or worsened hepatic encephalopathy [5,6]. Besides clinical symptoms/signs like esophageal bleedings, hepatic encephalopathy or ascites, Color Doppler ultrasound (CDUS) or computed tomography angiography (CTA) are routinely used for follow-up after TIPS [7,8,9]. According to recent guidelines commissioned by the Clinical Services and Standards Committee (CSSC) of the British Society of Gastroenterology in collaboration with the British Society of Interventional Radiology (BSIR) and British Association of the Study of the Liver (BASL), post-interventional CDUS is recommended one week after TIPS placement in patients prone for in-stent thrombosis or at risk for TIPS dysfunction. TIPS follow-up by CDUS is recommended at 6–12 months interval. Within HCC surveillance patients will be examined by CDUS biannually. Follow-up by invasive TIPS venography is indicated by the involved interventional radiologist in case of TIPS dysfunction is visualized by CDUS or in prothrombotic settings, e.g., in patients with Budd–Chiari-Syndrome [1]. A prior clinical trial demonstrated no beneficial impact of routine invasive venography 12 months after TIPS placement [10].

In addition to CDUS, including measurement of specific velocity criteria, contrast-enhanced ultrasound (CEUS) allows visualization of the TIPS perfusion in a real-time manner. So far, data about the role of CEUS for assessing TIPS perfusion are limited. In 2011, Micol et al. showed that CEUS can be a useful complement to CDUS. In 2020 Gao et al. reported high sensitivity and specificity of CEUS for evaluating TIPS anomalies with a high mechanical index [11,12]. Nevertheless, CEUS remains of minor importance in the follow-up management of TIPS and is rarely used as an additive imaging modality.

Prior studies could demonstrate the high diagnostic potential of CTA for assessing TIPS patency [13]. Ionizing contrast-enhanced CTA needs to be thoroughly evaluated in TIPS patients in case of concomitant renal insufficiency. One clinical trial elucidated the inferior diagnostic potential of magnetic resonance imaging (MRI) to investigate TIPS patency compared to CDUS and invasive portography [14].

For surgical treatment, a bare metal stent (BMS) or a (self-)expanded polytetraflouroethylene (ePTFE) stent can be selected. Differences are apparent by having significantly improved patency rates when using ePTFE and by having a reduced need for re-intervention compared to bare metal stents [15,16,17]. Therefore, the American Association for the Study of Liver Diseases (AASLD) recommended in their update in 2009 to prefer PTFE stents over bare metal stents [18]. In follow-up, a meta-analysis from 2019 showed an overall sensitivity of 96% in the detection of TIPS occlusion with a sensitivity of 100%. By comparing the performance of BMS and ePTFE, ePTFE showed a reduced sensitivity (82%) [19].

This retrospective single-center study aims at investigating the role of CEUS for TIPS follow-up.

## 2. Materials and Methods

Between 2008 and 2020, 106 patients were retrospectively enrolled in the study. The patient cohort consisted of 51 female and 55 male patients with an average age of 55.4 years and an age distribution of 23.3 to 82.1 years. The median age of the cohort was 55.1 years. Considering the male patients, the mean and median age was 56.1 years (age distribution: 30.2–82.1 years) and 55.2 years within the female cohort (age distribution: 23.3 to 80.8 years). Furthermore, over a maximum period of six months before and after performing CEUS, all available internal and external ultrasound, computed tomography (CT) and magnetic resonance imaging (MRI) examinations stored in the local achieving system were retrieved and evaluated. If TIPS revision was performed in the period between the CEUS examination and the other examinations (conventional ultrasound/CT/MRI), these examinations were not included in the detailed assessment. Thus, 92 conventional ultrasound examinations, 43 CT and 58 MRI examinations could be evaluated in comparison to the underlying CEUS examination. Within the 92 ultrasound examinations, 49 examinations were performed before and 43 after CEUS, within the 43 CT examinations 26 were before and 17 after and within the 58 MRI examinations 26 were before and 32 after CEUS.

Prior to study enrollment, a detailed medical consultation about all potential risks were conducted. Furthermore, oral and written informed consent was obtained by each of the 106 patients. The examination was performed in a supine position and the image material acquired was then transferred to the local achieving system for further analysis and detailed interpretation. Performance and interpretation of each study were carried out by a single experienced radiologist (EFSUMB level 3) with a professional experience of more than 20 years. All CEUS examinations were performed with high-end up-to-date ultrasound devices (Philips Ultrasound iU22, EPIQ 7, Seattle, Washington, DC, USA; Samsung RS 80, Seoul, Korea; GE Healthcare LOGIQ L9, Chicago, IL, USA; Siemens Ultrasound Sequoia, ACUSON Sequoia and S 2000, Mountain View, CA, USA). All included patients underwent native B-mode, CDUS and CEUS scans. To ensure a constant image impression, SonoVue^®^ (Bracco, Milan, Italy), approved by the U.S. Food and Drug administration (FDA), was used as a contrast agent in all included patients.

The specific feature of SonoVue^®^ as a second-generation blood-pool contrast agent is its purely intravascular distribution pattern. To prevent early destruction of the applied gas-filled microbubbles, a low mechanical index of <0.2 was used. The amount of contrast medium was between 1.0 and 1.2 mL of SonoVue^®^ with a subsequent application of 5–10 mL of 0.9% sodium chloride solution.

## 3. Results

In accordance with the inclusion criteria, 106 patients were enrolled in the study and 193 additional examinations, consisting of CDUS, CT and MRI were evaluated more closely with regard to accessibility and perfusion of the TIPS. All patients were referred to our Radiology Department for evaluating TIPS perfusion. During the examination, the TIPS was first visualized in native B-mode and CDUS, followed by an additional application of contrast agent for dynamic, non-invasive evaluation. None of the 106 included patients showed any kind of adverse effects related to the ultrasound contrast agent.

**Figure 1:**
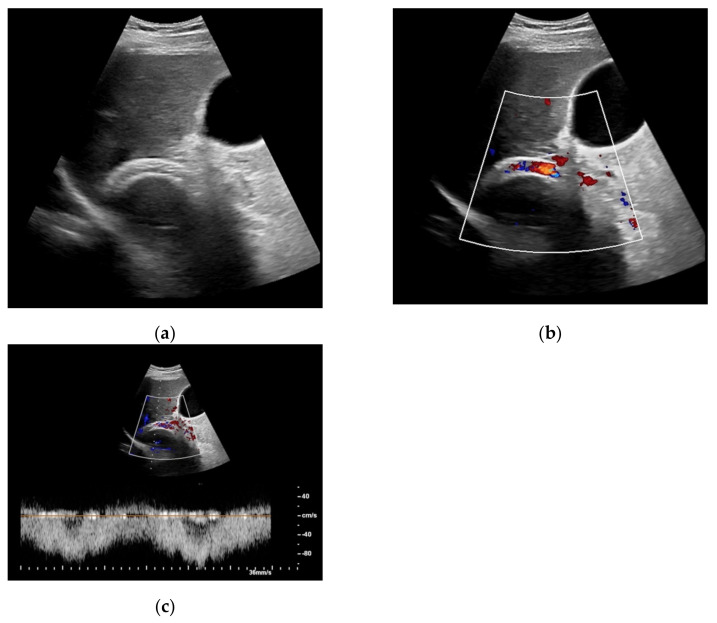
65-year-old patient with regular visualization of the transjugular intrahepatic portosystemic shunt (TIPS) during B-mode (**a**) and adequate visualization of the TIPS in Color Doppler ultrasound (**b**) with inconspicuous flow and flow velocity (**c**).

**Figure 2:**
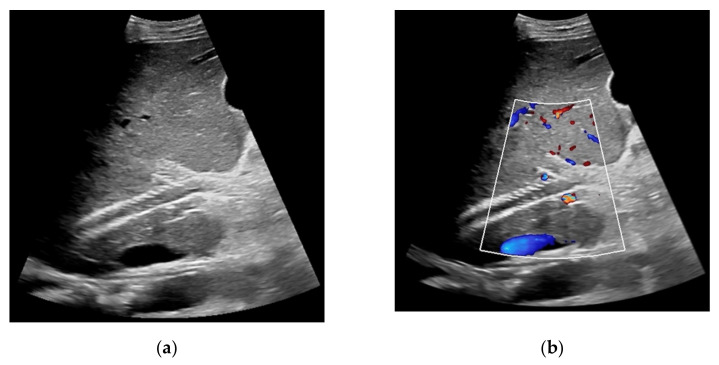
57-year-old female patient with occluded transjugular intrahepatic portosystemic shunt in B-mode (**a**) and consecutive absent flow signal in Color Doppler ultrasound (**b**).

**Figure 3:**
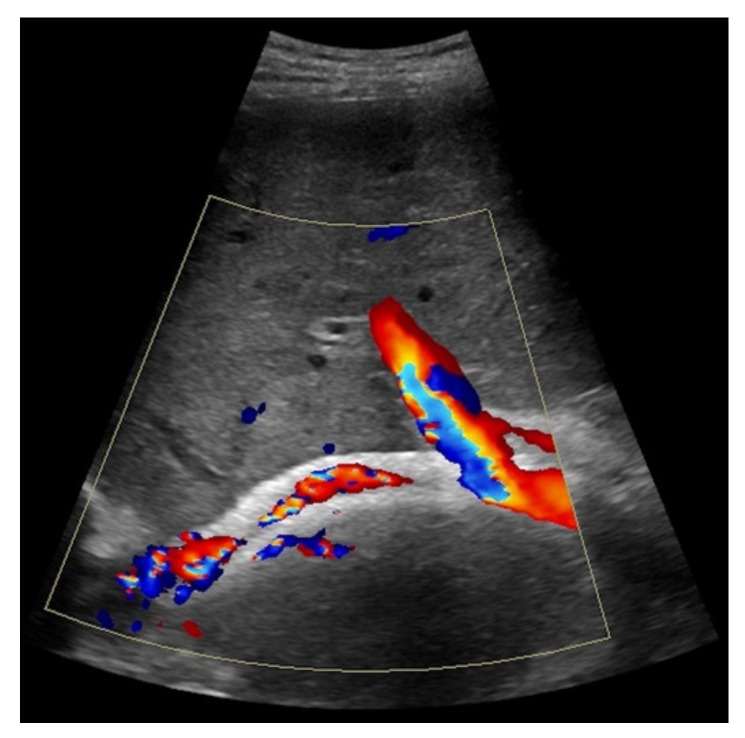
44-year-old female patient with inhomogeneous flow signal on Color Doppler ultrasound as a sign of stent thrombosis.

Based on initial findings from CDUS, 67% (*n* = 71) of the patients showed normal perfusion within the TIPS (Figure 1). 18% (*n* = 19) of the cases presented stent occlusion (Figure 2) while 6% of the patients (*n* = 6) had partial occluding stent thrombosis (Figure 3). The TIPS perfusion in five patients was only partially assessable, while in two patients an examination was not feasible due to extensive meteorism. The patients listed under “others” showed postoperative entrapped air between the wall of the vessel and the stent (*n* = 2) while one patient had two TIPS, one occluded and one with a normally detectable blood flow (Table 1).

**Figure 4:**
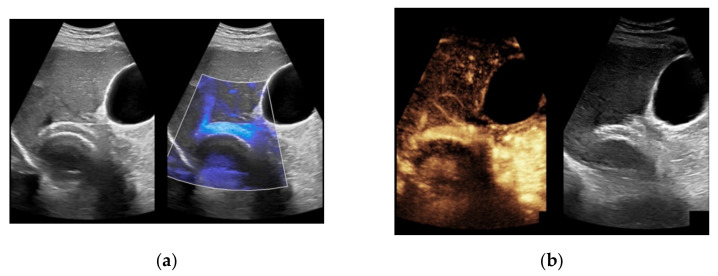

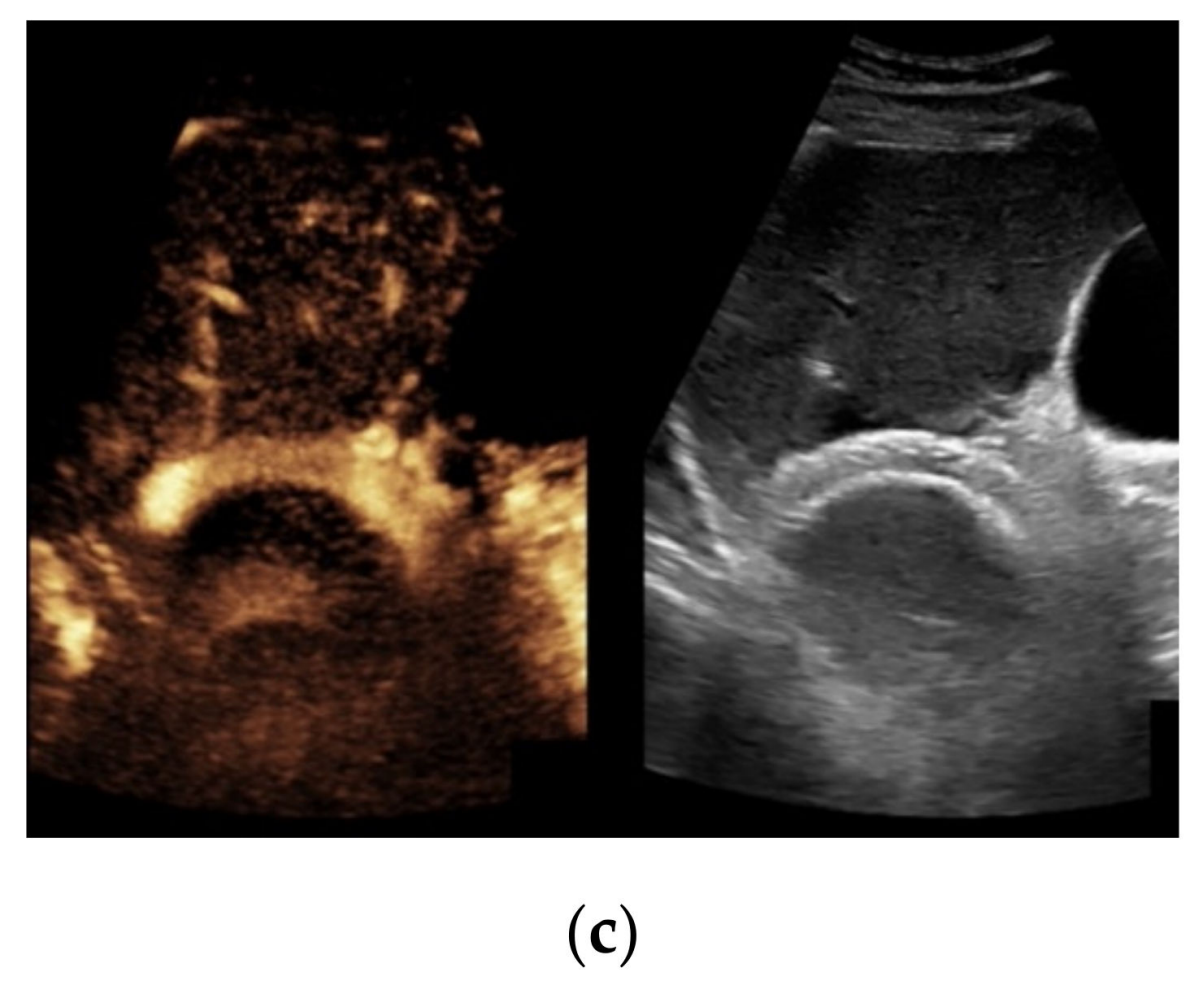
Same patient as in Figure 1 with continuous flow within the transjugular intrahepatic portosystemic shunt (TIPS) without thrombosis or occlusion using microflow imaging (**a**) and after administration of contrast agent (**b**,**c**).

**Figure 5:**
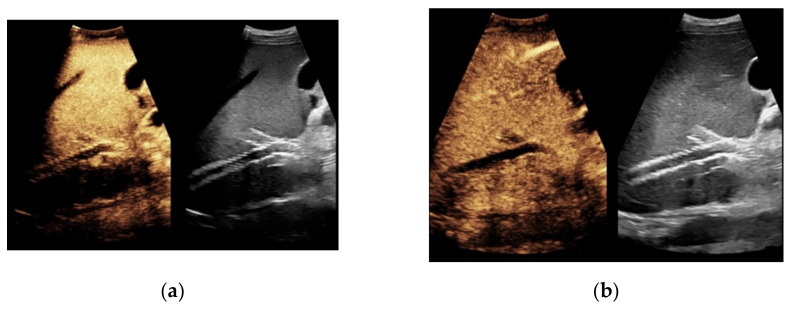
Same patient as in Figure 2. After administration of intravenous contrast, no intraluminal microbubbles can be registered within the transjugular intrahepatic portosystemic shunt compatible with complete occlusion (**a**,**b**).

**Figure 6:**
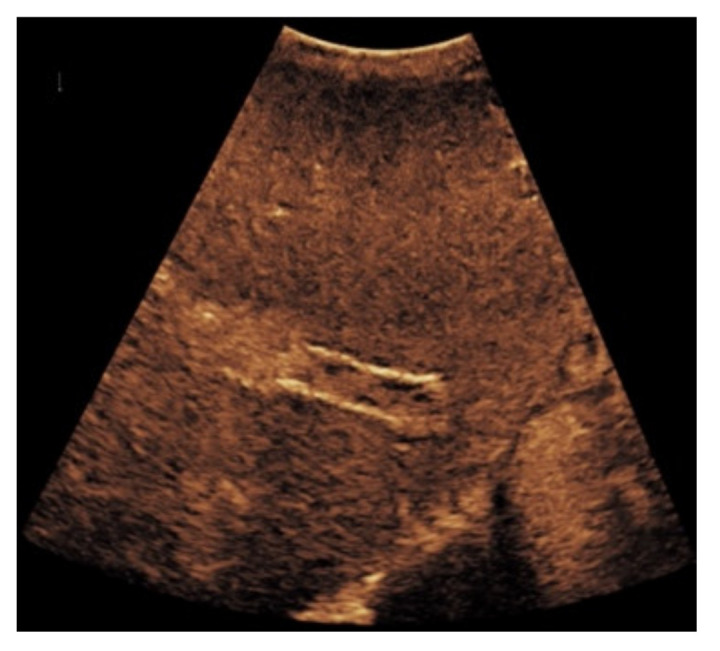
Same patient as in Figure 3. Contrast-enhanced ultrasound shows partial flow signal within the transjugular intrahepatic portosystemic shunt with marginal hypoechoic areas within the stent indicating thrombotic depositions.

When taking a closer look at the results of the CEUS examinations, 70.8% (*n* = 75) of the patients showed regular stent perfusion (Figure 4), 17.9% (*n* = 19) of the patients featured TIPS occlusion (Figure 5) while 6.6% (*n* = 7) patients had stent thrombosis (Figure 6). In contrast to the results from CDUS, there were no patients with only partial accessibility of the TIPS during CEUS, while the two patients with meteorism also were not assessable during CEUS. The three patients categorized in “others” showed simultaneous findings between CDUS and CEUS (Table 2).

**Figure 7:**
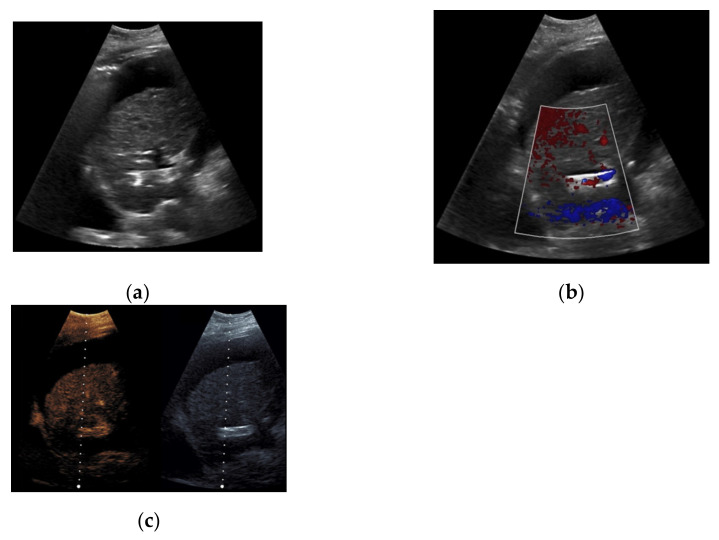
52-year-old female patient with irregular presentation of the transjugular intrahepatic portosystemic shunt (TIPS) on B-mode ultrasound (**a**) and partial flow on Color Doppler ultrasound (CDUS) (**b**). After contrast administration, in contrast to CDUS, regular and continuous contrast of the TIPS is seen without evidence of thrombotic alterations or occlusion (**c**).

Considering findings from CDUS and CEUS examination, in eight patients results were inconsistent (7.6%). Five patients who were only partially assessed via Color Doppler ultrasound showed a regular perfusion by using CEUS (Figure 7), two patients in whom inconspicuous TIPS perfusion was registered by CDUS showed partial occluding stent thrombosis by CEUS while in one patient suspected stent thrombosis in CDUS could not be verified by CEUS (Table 3).

Evaluating the additive examinations (CDUS, CT, MRT), the majority of cases showed high correlation to the results of the CEUS. In comparison with the results from CDUS, CEUS was found to be consistent in 96.7% of the cases, in comparison between CT and CEUS in 95.3% and in comparison between MRI and CEUS in 87.9% of the cases (Table 4, Table 5 and Table 6).

In comparison with CDUS, regular blood flow was visualized by CEUS in one patient while the TIPS was only partially visible by CDUS. One patient had normal blood flow during CDUS and a suspected stent thrombosis in CEUS and one patient had a normal blood flow in CEUS with suspected stent thrombosis during CDUS. The examinations of the three mentioned patients were between one and three months prior to the CEUS (Table 4).

In only two patients, findings from CEUS and CT were inconsistent. One patient was suspected to have partial stent thrombosis on CT, while total occlusion of TIPS could be demonstrated by CEUS. Another patient showed normal blood flow during CT, while stent thrombosis was detected during CEUS (Table 5).

If one evaluates the correlation between findings from MRI and CEUS, one finds the greatest discrepancy in contrast to the other comparative groups. Inconsistency was found in 12.1% of the cases. Four patients were not adequately assessed by MRI, whereby three TIPS were found to be inconspicuous and one was occluded in CEUS. The reason for the missing accessibility in the MRI was the marked ascites of the patients and the consequently limited image quality. One patient showed occlusion of the TIPS which could not be verified in CEUS due to meteorism. Two patients showed stent thrombosis during MRI while they showed a complete lack of blood flow during CEUS (Table 6).

## 4. Discussion

Transjugular intrahepatic portosystemic shunt displays an established percutaneous therapy for portal hypertension which requires adequate follow-up in order to assess stent perfusion and early detect alterations of stent perfusion, e.g., in case of stent thrombosis or stent occlusion. Color Doppler ultrasound can provide information about TIPS function based on velocity criteria, in particular the post-TIPS portosystemic gradient [7,20]. Due to its high sensitivity, direct transjugular venography is only recommended in case of pathological findings in the ultrasound examination or high probability of shunt dysfunction due to clinical worsening, e.g., recurrent ascites [1,21,22]. In general, there is no defined follow-up regime regarding imaging modality and timing after intervention [22,23]. Portal venography is often considered to be the gold standard in the diagnosis of TIPS dysfunction but is often put behind CDUS based on economic reasons, the invasive nature of the procedure and the associated radiation exposure [19,24]. So far, in asymptomatic as well as in symptomatic patients, CDUS is a commonly accepted screening modality usually performed 24 h, one, three and six months after intervention and then at 6-month intervals [22,25,26]. According to the recommendations of the AASLD in 2009, ePTFE stents are nowadays used in the majority of cases. However, this leads to limitations of the diagnostic validity in terms of follow-up. According to the 2019 meta-analysis by Manatsathit et al., assessing dysfunction of ePTFE TIPS stents by ultrasound showed a sensitivity of 82% with a specificity of only 58%. This indicates the importance of finding new approaches for diagnosing ePTFE stents with a comparable sensitivity and specificity as we have in BMS [19].

In the frame of vascular disorders, CEUS already proved to be superior to conventional sonography. For example, CEUS is superior to conventional ultrasound in the case of detecting endoleaks following endovascular aortic repair (EVAR) and, due to its high accuracy, can avoid supplementary contrast-enhanced CTs (CE-CT) in many cases [27,28,29]. Another diagnostic superiority of CEUS can be seen in the examination of the carotides, where CEUS already demonstrated higher sensitivity in the evaluation between occlusion and pre-occlusive stenosis compared to CDUS. In the latter example, CEUS showed comparable results to CT and MRI angiography [30,31,32,33].

In a previous clinical trial investigating TIPS perfusion by CEUS, Micol et al. showed concordance between CEUS and portography in 50 out of 56 cases. Among the remaining six cases not detected by CEUS, were two stent stenoses and four hepatic vein stenoses. With regard to the TIPS evaluation, two false-negative CEUS and 14 false negative CDUS examinations were present [11]. In a recent study from 2020, a substantial concordance of CEUS and portography was shown with a kappa value of 0.7396 (*n* = 16). The results further demonstrated that CEUS examinations at high MI showed tendencies for enhanced diagnostic performance compared to examinations at low MI. The authors argued one possible reason for the present finding was that at higher MI the microbubbles within the liver were more susceptible to be destroyed rapidly while the microbubbles within the stent were protected from acoustic pressure, thus resulting in a higher contrast between the surrounding liver tissue and TIPS could be achieved [12].

As demonstrated above, the contrast agent SonoVue^®^ was intravenously applied in every included patient for TIPS assessment. Nevertheless, any available ultrasound contrast agent may be used to evaluate TIPS perfusion. One possible contrast agent besides SonoVue^®^ is Sonazoid^®^ (Daiichi-Sankyo, Tokyo, Japan; GE Healthcare, Milwaukee, WI, USA). Sonazoid^®^ was first approved in Japan in 2007 and is now also available in Korea, Taiwan, Singapore and China. In Europe, so far it has only been approved in Norway. A special feature of Sonazoid^®^ is that the 2–3 µm microbubbles can be phagocytized by Kupffer cells in the liver, thereby generating a liver parenchyma-specific Kupffer phase which allows the investigator to evaluate the liver in a time frame of up to 60 min [34,35]. These benefits are of high value especially in terms of characterization of hepatic tumors such as hepatocellular carcinoma (HCC) [36,37]. Furthermore, its diagnostic value for hepatic metastasis was shown to be equivalent compared to that of other modalities such as PET-CT [38]. The Asian Federation of Societies for Ultrasound in Medicine and Biology (AFSUMB) expects in its 2020 published consensus letter on Recommendations for the Clinical Practice of Contrast-Enhanced Ultrasound using Sonazoid^®^ that due to the low rate of adverse events Sonazoid^®^ will also be approved in further European countries in the near future [39]. Due to the still pending approval of Sonazoid^®^ and the relevant higher costs compared to SonoVue^®^, SonoVue^®^ was used as the contrast agent of choice in the present study.

Referring to the recent scientific state of knowledge, we took a closer look at the results from CEUS with regard to stent dysfunction of TIPS. Comparing CDUS and CEUS, 98 of the 106 cases were concordant (Table 1 and Table 2). However, the eight patients with inconsistent findings showed relevant differences with a relevant effect on patient management. While TIPS was only partially visible in five patients by using CDUS, CEUS could visualize regular blood flow. Two other patients showed regular blood flow within TIPS using CDUS while following CEUS detected partial occluding stent thrombosis, and one patient with suspected stent thrombosis by CDUS showed regular blood flow in CEUS. This points out that in combination with CDUS, CEUS provides additional information that may prompt treatment changes. These results go in line with findings from Micol et al., in which a higher number of stenoses were identified by CEUS compared to CDUS [11]. With a 92.5% concordance to CDUS, CEUS proves its high sensitivity in follow-up. These results reflect the high diagnostic power which has already been described in the literature in other questions, such as the mentioned evaluation of endoleaks or coronary arteries.

Looking at the correlation of CEUS with CT and MRI, we see a high intermodal consistency. In comparison with corresponding CT images, 95.3% of the cases were concordant, whereas the concordance with MRI was only 87.9%. In contrast to findings from CT scans, there were disagreements in only two patients: one patient had a suspected stent thrombosis in the CT scan, a complete occlusion could be visualized by CEUS, one patient showed normal blood flow in the CT scan while stent thrombosis could be registered by CEUS. In the patient with a subsequent angiographically verified TIPS closure, 6 months elapsed between the initial CT and the follow-up CEUS. Considering the long period of time between the two examinations, the significance of this case is only limited. In the second patient, however, there were only 10 days between CT and CEUS, so an additive relevant finding could be assumed by using CEUS. By having a closer look at the correlation between MRI and CEUS, perfusion of the TIPS could not be assessed in three patients in the MRI, whereas these patients showed a regular blood flow when performing CEUS. These three cases, as well as another case which could only be assessed to a limited extent in the MRI and which showed complete occlusion in the CEUS, were due to pronounced ascites with a subsequently reduced imaging quality. Two patients were suspected to have a stent thrombosis in the MRI, while CEUS showed a complete occlusion. In both patients, the occlusion could be verified in CT angiography and both received a TIPS revision. Thus, analogous to CT and CDUS, additional findings could be identified, which were to some extent superior to the information provided by the other modalities.

Besides assessing shunt status, a relevant cohort of patients who underwent TIPS needs CDUS for HCC screening. In those patients, CEUS allows for dynamic TIPS evaluation; on the other hand, it may provide further sonomorphological information in case of indeterminate findings, e.g., in terms of HCC-suspicious lesions [40]. In a recent work, altered contrast-enhancement pattern of HCC lesions after TIPS insertion was described [41]. Moreover, a previous clinical trial described the development of focal nodular hyperplastic lesions in the liver of children who underwent TIPS insertion more than 3 years before. In this context, scrutinizing these lesions by means of CEUS would allow to rule out malignant origins [42], besides monitoring shunt function.

Consequently, CEUS can also provide advantages in view of the economic cost advantages in contrast to MRI and in view of the existing radiation exposure to CT. A further advantage of using CEUS compared to CT and MRI is its excellent safety profile, high tolerability of the contrast agent and the lack of a potential negative impact with regard to renal and thyroid gland function in CT and potential nephrogenic systemic fibrosis in case of impaired renal function in MRI [43,44,45]. None of the patients included in the study showed any kind of adverse effects related to the contrast agent. Despite the described advantages of CEUS for TIPS follow-up, it is still not widely used and only integrated in patient management at a few specialized centers. Up to date, there is no recommendation by the leading societies for using CEUS as imaging modality for monitoring TIPS.

Limitations of the present study comprise its retrospective nature and investigator dependency of CEUS examinations. In addition, evaluation of ePTFE stents within the first month after intervention is limited by artifacts and echo-reflection due to the graft material in both conventional ultrasound and CEUS [46].

## 5. Conclusions

In the follow-up regime after TIPS, CDUS is, next to clinical examination criteria, widely used in the diagnosis of shunt dysfunction or shunt occlusion. CEUS as a further non-invasive imaging modality may provide pivotal information in addition to CDUS, CT and MRI. CEUS is expedient due to its excellent safety profile, its direct accessibility and repeatability and its cost-effectiveness. By considering the increasing use ePTFE stents and the associated lower sensitivity and specificity of CDUS compared to BMS stents, especially in those patients, CEUS depicts a promising imaging modality for effective and safe TIPS follow-up.

## Figures and Tables

**Table 1:** Description of the patient cohort with regard to the results of Color Doppler ultrasound.

Color Doppler Ultrasound (Prior to Contrast-Enhanced Ultrasound)	Number of Patients	Percentage Value
Normal blood flow in TIPS	71	67.0%
Partial occluding stent thrombosis	6	5.7%
Occluded TIPS	19	17.9%
Partially visible	5	4.7%
Not evaluable	2	1.9%
Others	3	2.8%
Total	106	100%

**Table 2:** Description of the patient cohort with regard to the results of contrast-enhanced ultrasound.

Contrast-Enhanced Ultrasound	Number of Patients	Percentage Value
Normal blood flow in TIPS	75	70.8%
Partial occluding stent thrombosis	7	6.6%
Occluded TIPS	19	17.9%
Partially visible	0	0%
Not evaluable	2	1.9%
Others	3	2.8%
Total	106	100%

**Table 3:** Depiction of the different findings during Color Doppler ultrasound and contrast-enhanced ultrasound.

Color Doppler Ultrasound (Prior to Contrast-Enhanced Ultrasound) vs. Contrast-Enhanced Ultrasound	Number of Patients	Percentage Value
partially visible ➔ normal blood flow	5	4.7%
normal blood flow ➔ suspected stent thrombosis	5	1.9%
suspected stent thrombosis ➔ normal blood flow	1	0.9%
consistent findings	98	92.5%
Total (*n* = 106)	8	7.6%

**Table 4:** Depiction of the different findings during Color Doppler ultrasound (6 months before or after contrast-enhanced ultrasaound (CEUS)) and CEUS.

Color Doppler Ultrasound (6 Months before or after Contrast-Enhanced Ultrasound) vs. Contrast-Enhanced Ultrasound	Number of Patients	Percentage Value
partially visible ➔ normal blood flow	1	1.1%
normal blood flow ➔ suspected stent thrombosis	1	1.1%
suspected stent thrombosis ➔ normal blood flow	1	1.1%
consistent findings	89	96.7%
Total (*n* = 92)	3	3.3%

**Table 5:** Depiction of the different findings during computed tomography and contrast-enhanced ultrasound.

Computed Tomography (6 Months before or after Contrast-Enhanced Ultrasound) vs. Contrast-Enhanced Ultrasound	Number of Patients	Percentage Value
suspected stent thrombosis ➔ occluded TIPS	1	2.3%
normal blood flow ➔ suspected stent thrombosis	1	2.3%
consistent findings	41	95.3%
Total (*n* = 43)	3	4.7%

**Table 6:** Depiction of the different findings during Magnetic Resonance Imaging and contrast-enhanced ultrasound.

Magnetic Resonance Imaging (6 Months before or after Contrast-Enhanced Ultrasound) vs. Contrast-Enhanced Ultrasound	Number of Patients	Percentage Value
Not evaluable ➔ normal blood flow	3	5.2%
Limited assessment (ascites) ➔ occluded TIPS	1	1.7%
Occluded TIPS ➔ not visible (meteorism)	1	1.7%
Suspected stent thrombosis ➔ occluded TIPS	2	3.4%
Consistent findings	51	87.9%
Total (*n* = 58)	7	12.1%

## Data Availability

The data presented in this study are available on request from the corresponding author. The data are not publicly available due to privacy.
